# Improving Accuracy of Brainstem MRI Volumetry: Effects of Age and Sex, and Normalization Strategies

**DOI:** 10.3389/fnins.2020.609422

**Published:** 2020-12-23

**Authors:** Laura Sander, Antal Horvath, Simon Pezold, Simon Andermatt, Michael Amann, Tim Sinnecker, Maria J. Wendebourg, Eva Kesenheimer, Özgür Yaldizli, Ludwig Kappos, Cristina Granziera, Jens Wuerfel, Philippe Cattin, Regina Schlaeger

**Affiliations:** ^1^Neurologic Clinic and Policlinic, Departments of Medicine and Clinical Research, University Hospital Basel and University of Basel, Basel, Switzerland; ^2^Translational Imaging in Neurology (ThINK) Basel, Department of Biomedical Engineering, University of Basel, Basel, Switzerland; ^3^Department of Biomedical Engineering, Center for Medical Image Analysis & Navigation (CIAN), University of Basel, Allschwil, Switzerland; ^4^Department of Biomedical Engineering, Medical Image Analysis Center (MIAC AG) and qbig, University of Basel, Basel, Switzerland

**Keywords:** brainstem, covariate, normalization, segmentation, volumetry, MD-GRU

## Abstract

**Background:** Brainstem-mediated functions are impaired in neurodegenerative diseases and aging. Atrophy can be visualized by MRI. This study investigates extrinsic sources of brainstem volume variability, intrinsic sources of anatomical variability, and the influence of age and sex on the brainstem volumes in healthy subjects. We aimed to develop efficient normalization strategies to reduce the effects of intrinsic anatomic variability on brainstem volumetry.

**Methods:** Brainstem segmentation was performed from MPRAGE data using our deep-learning-based brainstem segmentation algorithm MD-GRU. The extrinsic variability of brainstem volume assessments across scanners and protocols was investigated in two groups comprising 11 (median age 33.3 years, 7 women) and 22 healthy subjects (median age 27.6 years, 50% women) scanned twice and compared using Dice scores. Intrinsic anatomical inter-individual variability and age and sex effects on brainstem volumes were assessed in segmentations of 110 healthy subjects (median age 30.9 years, range 18–72 years, 53.6% women) acquired on 1.5T (45%) and 3T (55%) scanners. The association between brainstem volumes and predefined anatomical covariates was studied using Pearson correlations. Anatomical variables with associations of |*r*| > 0.30 as well as the variables age and sex were used to construct normalization models using backward selection. The effect of the resulting normalization models was assessed by % relative standard deviation reduction and by comparing the inter-individual variability of the normalized brainstem volumes to the non-normalized values using paired t- tests with Bonferroni correction.

**Results:** The extrinsic variability of brainstem volumetry across different field strengths and imaging protocols was low (Dice scores > 0.94). Mean inter-individual variability/SD of total brainstem volumes was 9.8%/7.36. A normalization based on either total intracranial volume (TICV), TICV and age, or v-scale significantly reduced the inter-individual variability of total brainstem volumes compared to non-normalized volumes and similarly reduced the relative standard deviation by about 35%.

**Conclusion:** The extrinsic variability of the novel brainstem segmentation method MD-GRU across different scanners and imaging protocols is very low. Anatomic inter-individual variability of brainstem volumes is substantial. This study presents efficient normalization models for variability reduction in brainstem volumetry in healthy subjects.

## Introduction

The brainstem as the anatomical and functional link between the cerebrum, the cerebellum, and the spinal cord is a vitally important structure, playing a key role in controlling respiratory and cardiac function, defense reflexes, and awareness. From cranial to caudal, the brainstem is divided into the three substructures mesencephalon, pons, and medulla oblongata. It carries white matter tracts to and from the cerebrum, the spinal cord, and the cerebellum, multiple cranial nerves and reticular nuclei (Nieuwenhuys, 1985; Naidich et al., 2009). While the mesencephalon plays an important role mainly in oculomotor, optic, and acoustic function, the pons contains important white matter tracts as well as cranial nerve nuclei for facial sensory and motor functions (Basinger and Hogg, 2020). The medulla oblongata regulates respiratory function and contains important reflex centers e.g., for coughing and swallowing (Bolser et al., 2015; Ikeda et al., 2017).

Studying the brainstem is crucial for our understanding of both physiologic neurological function and neurological diseases.

Physiologic aging is associated with morphological changes throughout the brain, especially with atrophy of the cortex, deep gray matter structures (Walhovd et al., 2011), and the cerebellum (Woodruff-Pak et al., 2010). Brainstem-mediated functions such as cardiovascular reflexes (Vita et al., 1986), swallowing (Sura et al., 2012), gaze stability (Anson et al., 2016), and auditory function (Skoe et al., 2015) can be altered with increasing age.

Several neurodegenerative diseases as e.g., Alzheimer's (Grinberg et al., 2009) and Parkinson's disease (Grinberg et al., 2009), progressive supranuclear palsy (Williams and Lees, 2009), multisystem atrophy (Ghorayeb et al., 2002), amyotrophic lateral sclerosis (Warabi et al., 2017), and multiple sclerosis (Noseworthy et al., 2000) can affect the brainstem or its substructures. Preferential involvement of distinct brainstem substructures has been observed in several neurodegenerative diseases e.g., mesencephalic atrophy in patients with progressive supranuclear palsy (Cosottini et al., 2007). Progressive degeneration of brainstem structures in these diseases can result in autonomic dysfunction, dysphagia, dysarthria, and other symptoms interfering with the patient's quality of life and potentially also survival (Grinberg et al., 2010; Kim et al., 2018).

Brainstem tissue loss – acquired either during aging or neurodegenerative diseases – can be visualized and quantified by MRI *in vivo*, offering a potential as diagnostic, prognostic, or therapeutic marker in these diseases.

A recently published deep-learning-based algorithm provided accurate, highly reproducible, and robust brainstem segmentation in healthy subjects (HS) and patients with Alzheimer's disease and multiple sclerosis (Andermatt et al., 2016, 2018; Sander et al., 2019).

Despite its successful application in patients, volumetry of the brainstem and its substructures has not yet been systematically assessed in HS. Brainstem volume assessments are subject to inter-individual variation, e.g., due to head size, head position in the scanner, sex or body height. Volume normalization reduces the physiologic inter-individual measurement variation due to individual anatomical effects, ideally without interfering with measurements related to possible disease processes. This allows for better statistical comparison between two inhomogeneous groups, such as healthy controls and patients. A frequently used normalization parameter for brain volumes is the FreeSurfer-derived total intracranial volume (TICV; Whitwell et al., 2001) and SIENAX-derived volumetric scaling factor (v-scale; Fein et al., 2004). So far, normalization covariates for brainstem segmentation have not yet been investigated and relevant normalization factors for brainstem volumetry are not known.

Using a novel fully-automated deep-learning-based segmentation approach, the objectives of this study were to assess:

a) the extrinsic variability of brainstem volumes depending on different scanners, field strengths, and acquisition protocols,
b) the intrinsic anatomical variability and the influence of age and sex on the brainstem and its substructure volumes in HS, and
c) the effects of normalization models on variability in brainstem volumetry.

## Materials and Methods

### Brainstem Segmentation

Brainstem volumes were assessed using a recently published fully-automated segmentation approach based on multi-dimensional gated recurrent units (MD-GRU). The deep-learning-based algorithm provides accurate, robust, and reproducible segmentations of the brainstem and its substructures (Andermatt et al., 2016, 2018; Sander et al., 2019). All segmentations were visually inspected.

### Image Acquisition for Assessing the Extrinsic Variability of Brainstem Volumes by Different Scanners and Protocols

Eleven HS (mean age 36.9 years, median age 33.3 years, range 24–56 years, SD 11.2, 7 women) were scanned twice within the same day on two different scanners (Magnetom Prisma and Skyra, Siemens Healthineers, Erlangen, Germany). Acquisition parameters for all scans were TR = 2,300 ms, TI = 900 ms, TE = 3 ms, α = 9°, spatial resolution of 1 × 1 × 1mm^3^. Brainstem volumes were obtained from 3D high-resolution T1w MR imaging data (MPRAGE).

Additional 22 HS (mean age 29.7 years, median age 27.6 years, range 20–51 years, SD 8.1, 50% women) were assessed with two different MR protocols, each on the same 1.5T Magnetom Avanto scanner (Siemens Healthineers, Erlangen, Germany). Acquisition parameters of the MPRAGE scans for the first protocol were TR = 2,700 ms, TI = 950 ms, TE = 5 ms, α = 8°, spatial resolution of 1 × 1 × 1 mm^3^, for the second protocol TR = 2,080 ms, TI = 1,100 ms, TE = 3 ms, α = 15°, spatial resolution of 0.98 × 0.98 × 1 mm^3^. Brainstem segmentation volumes were obtained for the two protocols.

Finally, MPRAGE images of the same 22 HS acquired on a 1.5T Avanto (acquisition parameters TR = 2,080 ms, TI = 1,100 ms, TE = 3 ms, α = 15°, spatial resolution of 0.98 × 0.98 × 1 mm^3^) and in addition on a 3T Skyra (TR = 2,300 ms, TI = 900 ms, TE = 3 ms, α = 9°, spatial resolution of 1 × 1 × 1 mm^3^, parallel imaging, acceleration factor 2) were analyzed to compare brainstem segmentations from both assessments.

### Image Acquisition for Assessing the Intrinsic Anatomical Variability

3D high-resolution T1weighted MR imaging data (MPRAGE) were obtained from 110 HS (mean age 34.9 years, median age 30.9 years, range 18–72 years, SD 12.8, 53.6% women) on 1.5T (45%) and 3T (55%) scanners with two different protocols, respectively.

Briefly, acquisition parameters for the 1.5T Magnetom Avanto scanner (Siemens Healthineers, Erlangen, Germany) were TR = 2,080 ms, TI = 1,100 ms, TE = 3 ms, α = 15°, spatial resolution of 0.98 × 0.98 × 1 mm^3^ (Bendfeldt et al., 2009; Weier et al., 2014) and TR = 2,700 ms, TI = 950 ms, TE = 5 ms, α = 8°, spatial resolution of 1 × 1 × 1 mm^3^. For the 3T Prisma scanner (Siemens Healthineers, Erlangen, Germany) acquisition parameters were TR = 1,680 ms, TI = 900 ms, TE = 2.5 ms, α = 8°, spatial resolution of 1 × 1 × 1 mm^3^ and TR = 2,300 ms, TI = 900 ms, TE = 3 ms, α = 9°, spatial resolution of 1 × 1 × 1 mm^3^, acceleration factor 2.

Written informed consent was obtained from all participants mentioned above.

All brainstem segmentations were visually inspected for anatomic accuracy.

### Statistical Analyses

Statistical analyses were performed using JMP Pro 14 and SPSS 25.

#### Assessing the Extrinsic Variability of Brainstem Volumes

Dice coefficients were each calculated comparing brainstem segmentations obtained in the same individual (a) on different scanners (1.5T vs. 3T), (b) on the same scanner but with different protocols, and (c) on different scanners and protocols.

#### Assessing the Intrinsic Variability of Brainstem Volumes

To assess the inter-individual variability, the respective deviation from the group mean was calculated for each subject as (measured volume – mean volume)/mean volume.

#### Age and Sex Effects

Differences in brainstem/brainstem substructure volumes between men and women were assessed using linear regression analysis with (a) age as covariate, as well as in a sensitivity analysis with (b) field strength, (c) acquisition protocol, and (d) TICV as additional covariates, respectively. Correction for multiple testing (4 analyses) was performed using the Bonferroni correction, adjusting the level of significance to *p* <0.05/4.

The associations between age and brainstem/brainstem substructure volumes were assessed using linear regression analysis covarying for sex. Differences of brainstem volumes in younger vs. older persons (below vs. above the group mean) were assessed using linear regression analysis with (a) field strength and (b) acquisition protocol as additional covariates, respectively.

#### Assessing Effects of Different Normalization Models on Reduction of Anatomical Variability

**Figure 1:**
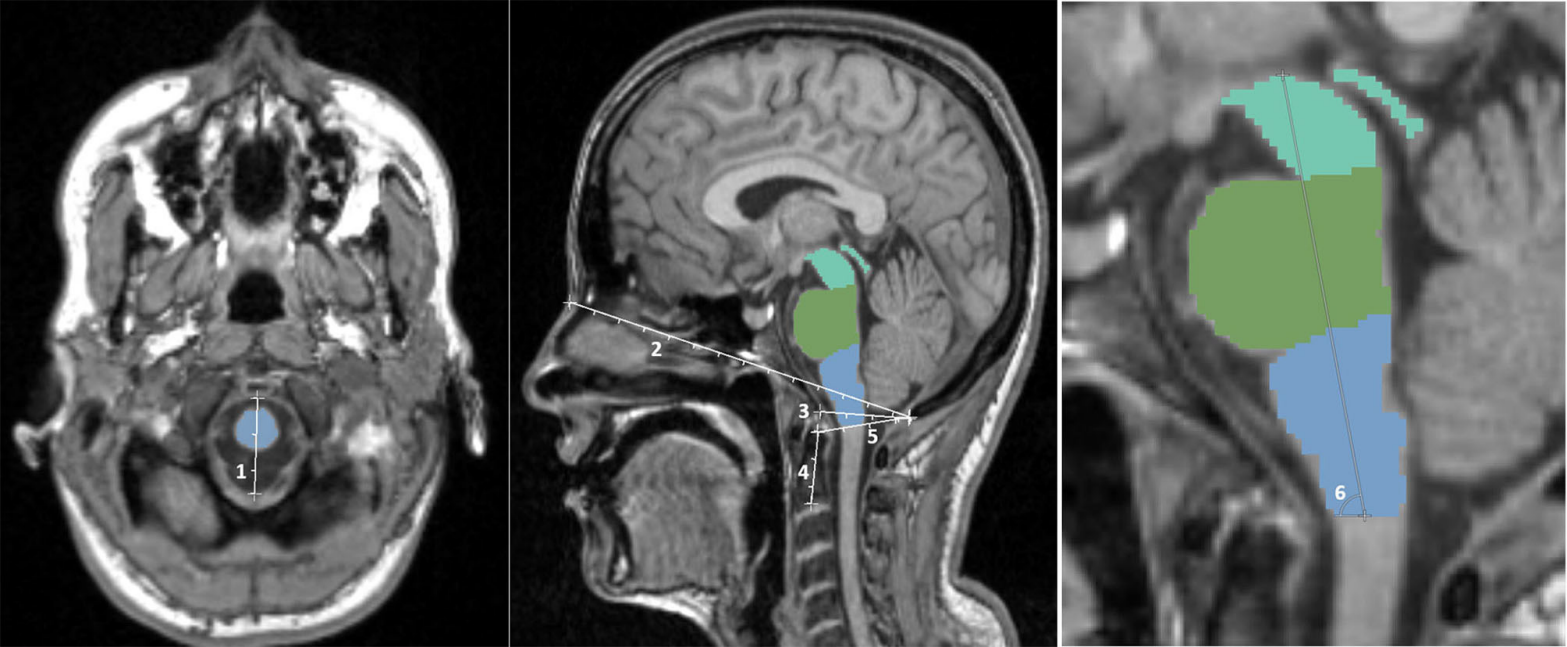
Illustration of the skull-based parameters foramen magnum diameter (1), nasion-opisthion (2), basion-opisthion (3), dens length (4), dens-opisthion (5), and brainstem angle (6).

Several skull-derived and intracerebral anatomical structures were assessed regarding their potential role as covariates for brainstem volumes. As skull-based parameters, foramen magnum diameter, nasion-opisthion, basion-opisthion (Mc Rae's line), dens length, dens-opisthion, and brainstem angle were assessed (Figure 1) using the open-source software 3D Slicer 4.8.1 (www.slicer.org) and GIMP (www.gimp.org). Additionally, v-scale by SIENAX (http://www.fmrib.ox.ac.uk/analysis/research/siena), TICV, gray matter (GM), and white matter (WM) volumes, as well as total brain volume (BV) by FreeSurfer (http://surfer.nmr.mgh.harvard.edu/) were determined.

The associations of these parameters with brainstem volume were first assessed using Pearson correlation coefficients. To correct for multiple tests, Bonferroni correction was performed with a correction factor of *n* = 12 (11 anatomical variables and age) (*p* <0.05/12).

Only those anatomical metrics showing a significant association with all brainstem and brainstem substructure volumes with a Pearson correlation coefficient of |*r*| > 0.30 (Cohen, 1988), respectively, were considered as potential normalization covariates in further analyses.

We then performed a backward selection procedure starting with a model with total brainstem volume as outcome parameter, and all anatomical variables with a Pearson correlation coefficient of |*r*| > 0.30 in univariate analysis as well as age and sex as predictor variables.

This procedure was performed (a) with TICV and (b) with v-scale separately, as these parameters are co-linear.

The adjusted *r*^2^ of the models resulting from the backward selection were reported, as well as of a further simplified model (considering simple application with preference for fewer and easy to measure covariates).

The normalization of brainstem volumes and its substructure volumes was then performed by using the following equation (Sanfilipo et al., 2004; Papinutto et al., 2019):

$$\begin{array}{l} {\text{Volume}}_{\text{predicted}}={\text{Volume}}_{\text{measured}}+\text{a}\left({\text{X}}_{\text{mean}}-{\text{X}}_{\text{measured}}\right)\\ +\text{b}\left({\text{Y}}_{\text{mean}}-{\text{Y}}_{\text{measured}}\right)+\text{c}\left({\text{Z}}_{\text{mean}}-\;{\text{Z}}_{\text{measured}}\right) \end{array}$$

with a, b, c being the estimates (regression coefficients) obtained by the linear regression analysis and X, Y, Z their measured values.

To assess the performance of the different normalization models, the inter-individual variability of the normalized brainstem volumes of each model was first compared to the variability of the non-normalized brainstem volumes using paired-*t*-tests, with Bonferroni correction for multiple tests (3 models, *p* <0.05/3).

In a second step, we compared the performance between the normalization models by comparing the inter-individual variability of the normalized brainstem volumes by a one-way ANOVA (analysis of variance).

The performance of the different normalization models was also expressed by the % relative standard deviation (%RSD) reductions of the predicted volumes to the %RSD of the non-normalized, measured volumes of the whole group (*n* = 110). The relative standard deviation (RSD) is the standard deviation divided by the mean volume.

## Results

### Brainstem Segmentation

The automated brainstem segmentation approach yielded anatomically accurate results in all subjects in <200 s/scan on an NVidia GeForce GTX 1080 GPU: All obtained brainstem segmentations were considered anatomically correct in its location and borders, when visually inspected, no manual correction was needed.

### Assessing the Influence of Different Scanners and Protocols on Brainstem Volume Variability

The results of brainstem segmentation comparisons from different scanners and protocols are shown in Table 1.

**Table 1:** Mean Dice scores, SD and 95%CI for the total brainstem, mesencephalon, pons, and medulla oblongata volumes comparing segmentations of the same individuals obtained from different scanners, different protocols, and different scanners and protocols.

	**Comparison of different scanners**	**Comparison of different protocols**	**Comparison of different scanners and protocols**
**Brainstem**			
Mean dice score SD 95%CI	0.9736 0.004 0.9710–0.9762	0.9685 0.003 0.9670–0.9699	0.9737 0.002 0.9730–0.9743
**Mesencephalon**			
Mean dice score SD 95%CI	0.9567 0.009 0.9516–0.9618	0.9501 0.007 0.9473–0.9529	0.9577 0.005 0.9557–0.9597
**Pons**			
Mean dice score SD 95%CI	0.9756 0.003 0.9736–0.9776	0.9747 0.002 0.9737–0.9757	0.9760 0.002 0.9753–0.9767
**Medulla oblongata**			
Mean dice score SD 95%CI	0.9496 0.013 0.9419–0.9573	0.9362 0.012 0.9311–0.9413	0.9525 0.007 0.9495–0.9554

MD-GRU derived brainstem segmentations from scans of the same individual obtained on different 3T scanners (Prisma vs. Skyra) using the same acquisition protocol showed Dice scores between 0.95 and 0.98.

Similarly, Dice coefficients comparing segmentations from scans of the same individual using different imaging protocols as specified above on the same 1.5T Avanto scanner were between 0.94 and 0.97.

Dice coefficients of segmentation comparisons using different scanners (1.5T Avanto vs. 3T Skyra) and different protocols were similarly high (0.95–0.98).

### Intrinsic Anatomical Variability

#### Inter-individual Variability of Brainstem Segmentations

Mean % inter-individual variability/SD for the different brainstem volumes were: 9.1/6.40 (mesencephalon), 11.2/8.01 (pons), 10.0/7.23 (medulla oblongata) and 9.8/7.36 (total brainstem).

#### Age and Sex Influence

Men had significantly larger unadjusted volumes of the total brainstem, mesencephalon, pons, and medulla oblongata (all *p* <0.0001, respectively), compared to women with total brainstem volumes of 28274.0/2670.1 for men (mean [mm^3^]/SD) vs. 24826.2/2824.1 (mean [mm^3^]/SD) for women. However, after adjustment for age and TICV (to account for head size differences), men showed significantly larger medulla oblongata volumes compared to women (Appendix A in Supplementary Material) with all other comparisons being insignificant after Bonferroni correction. Adjustment for field strength or protocol did not alter these observations.

With adjustment for sex, there was no significant association between age and total brainstem (*p* = 0.4131) volumes. In line with this observation, total brainstem volumes did not differ significantly between older subjects (aged above the group mean of 35 years; *n* = 44) and younger subjects (<35 years; *n* = 66) (*p* = 0.3068) with adjustment for sex. Results were comparable for mesencephalon, pons, and medulla oblongata volumes (Appendix B in Supplementary Material).

This finding was independent of additional adjustment for field strength or acquisition protocol (Appendix C in Supplementary Material).

#### Assessing Potential Normalization Models for Anatomical Variability Reduction

Table 2 reports the strength of the correlations of total brainstem volume with each of the investigated variables. Amongst these metrics, nasion-opisthion, dens length, TICV, v-scale, WM, GM, and BV (all normally distributed) showed a significant correlation with brainstem and all substructure volumes surviving the Bonferroni correction for multiple tests with a Pearson correlation coefficient |*r*| > 0.30 (Table 2 and Appendix D in Supplementary Material) and are therefore potential univariate predictors. As BV, GM, and WM volumes can be altered by neurodegenerative processes, these variables were not considered in further analyses.

**Table 2:** Pearson correlation coefficients of the metrics and volumes of the total brainstem.

	**Brainstem Volume**	
**Variable**	***p***	**Pearson correlation coefficient**
Nasion-opisthion	**<** **0.0001**	0.462
Dens length	**<** **0.0001**	0.388
TICV	**<** **0.0001**	0.748
v-scale	**<** **0.0001**	−0.747
Age	0.213	0.120
WM volume	**<** **0.0001**	0.788
GM volume	**<** **0.0001**	0.559
BV	**<** **0.0001**	0.723
Basion-opisthion	0.0139	0.234
Foramen magnum diameter	0.5978	0.051
Dens-opisthion	**0.0006**	0.324
Brainstem angle	0.0112	0.241

*p-values surviving the Bonferroni correction (for 12 tests) <0.0042 are bolded. TICV (total intracranial volume), WM (white matter), GM (grey matter), BV (brain volume)*.

Pearson correlation coefficients of all variables and brainstem substructure volumes are shown in Appendix D in Supplementary Material.

#### Comparison of Different Normalization Models

The backward selection procedure resulted in two models based on TICV and age (Model 1a) and v-scale (Model 2). The model based on TICV and age was further simplified to TICV alone (Model 1b) (Table 3).

**Table 3:** Linear regression analysis with total brainstem volumes as outcome and normalization variables after backward selection.

	**Brainstem**		
	***p***	**Adj. *r*^**2**^**	**Estimate**
**Model 1a**	<0.0001	0.586	
TICV	<0.0001		0.0149
Age	0.0036		46.7822
**Model 1b**	<0.0001	0.556	
TICV	<0.0001		0.0146
**Model 2**	<0.0001	0.553	
v-scale	<0.0001		−17,650.89

Results of the linear regression analysis with brainstem substructure volumes as outcome are shown in Appendix E in Supplementary Material.

The model with TICV and age consistently yielded the highest adjusted *r*^2^. However, eliminating the variable age from the Model 1a did not substantially reduce the variance explained. Brainstem volume normalization by TICV (Model 1b) or v-scale (Model 2) yielded comparably high *r*^2^.

Efficiency of the normalization based on Models 1a, 1b, and 2 is reported as the %RSD, the relative %RSD reduction, and the mean % inter-individual variability achieved by the respective models with respect to measured brainstem volumes (Table 4). Normalization by Models 1a, 1b, 2 yielded similarly efficient relative %RSD reductions, with a reduction of 33–36% for total brainstem volumes and up to 46% for mesencephalon volumes. Normalization effects on mesencephalon, pons, and medulla oblongata volumes are reported in Appendix F in Supplementary Material.

**Table 4:** % relative standard deviation (RSD, standard deviation divided by the mean volume), relative %RSD reduction, mean % inter-individual variability [(measured volume – mean volume)/mean volume], and SD of the % inter-individual variability with respect to the measured total brainstem volumes for normalizations based on Models 1a, 1b, and 2.

	**Non-normalized**	**Model 1a**	**Model 1b**	**Model 2**
**Brainstem**				
%RSD	12.26	7.82	8.13	8.16
Relative %RSD reduction [%]		36.22	33.69	33.44
Mean % inter-individual variability	9.76	6.26	6.55	6.43
SD	7.36	4.64	4.78	4.98

The total brainstem volume normalization based on each of the three models significantly reduced the inter-individual variability compared to the non-normalized brainstem volumes (p/ 95% CI of the variability difference for Model 1a *p* <0.0001/[0.022; 0.048], Model 1b *p* <0.0001/[0.019; 0.046], Model 2 *p* <0.0001/[0.007; 0.020]) surviving the Bonferroni correction (<0.05/3), respectively. One-way ANOVA comparing the inter-individual variability across all models showed no significant difference between different models for total brainstem (*F*_2, 327_ = 0.099, *p* = 0.905), mesencephalon (*F*_2, 327_ = 0.631, *p* = 0.533), pons (*F*_2, 327_ = 0.115, *p* = 0.892), and medulla oblongata (*F*_2, 327_ = 0.187, *p* = 0.829) volumes.

## Discussion

Using a novel, accurate, fully automated, and rapid brainstem segmentation method (Sander et al., 2019) we explored sources of extrinsic (field strength, protocol) as well as intrinsic anatomical variability, investigated age and sex influences on brainstem volumes on high-resolution MPRAGE images in HS and developed potential normalization strategies for variability reduction in brainstem volumetry.

The extrinsic variability of our brainstem volumetry assessment method with respect to different acquisition protocols, hardware, and magnetic field strength was low; the comparisons of brainstem segmentations obtained in the same individuals assessed by different scanners as well as different protocols and both different scanners and protocols yielded very high Dice scores (≥0.94). These results confirm the robustness of the applied brainstem segmentation algorithm with respect to different image acquisition settings, i.e., different scanners with 1.5T and 3T field strength and different acquisition protocols.

Consistent with previous studies, our results showed no relevant age dependent volume reduction of the brainstem and its substructures in this cohort aged between 18 and 72 years. With a mean age of 34.9 years and a median age of 30.9 years, this cohort might be, however, more representative for middle-aged and younger adults. Based on the result we cannot fully exclude a decline in brainstem volume in healthy persons of advanced age.

The lack of an age dependent volume reduction observed in this cohort is consistent with previous studies: Several cross-sectional brainstem segmentation studies based on manual brainstem segmentation reported no association of ventral pons volumes with age (Raz et al., 2001; Sullivan et al., 2004). Likewise, no age effects were found in total brainstem and medulla oblongata volumes (Luft et al., 1999; Lee et al., 2009). Lambert et al. (2013) found isolated midbrain atrophy in HS of age older than 60 years, predominantly due to a volume loss of the superior cerebellar fiber bundles which are not taken into account in our mesencephalon volumetry definition.

In our study, men showed significantly larger unadjusted volumes of the brainstem and its substructures compared to women, which is in line with findings by Raz et al. (2001) and Sullivan et al. (2004). Lee et al. (2009) also reported larger medulla oblongata volumes in men. However, after adjustment for TICV (to account for head size differences) and age, the differences observed between men and women remained only significant for medulla oblongata volumes.

Anatomical variations between HS are an important source of brainstem volume variability with this cohort showing an inter-individual variability of about 10% for brainstem volumes. Therefore, normalization of brainstem volumes is crucial to reduce measurement variation to facilitate the applicability of brainstem volumetrics as a surrogate marker for prognosis, disease course monitoring and therapeutic monitoring in neurodegenerative diseases as e.g., amyotrophic lateral sclerosis, Alzheimer's and Parkinson's disease.

Intracerebral metrics like GM, WM, and BV are expected to be altered by neurodegenerative pathologies, and their potential use as covariates of brainstem volumes might therefore only be adequate in studies involving HS. Hence these parameters were not considered as adequate brainstem normalization parameters.

Models based on FreeSurfer-derived TICV and SIENAX-derived v-scale, two commonly used normalization parameters, as well as TICV and age scored highest adjusted *r*^2^ in linear regression analyses with brainstem and brainstem substructures as outcomes and were therefore further tested as normalization variables.

Normalization for anatomic variation of head size by TICV and age reduced the %RSD of total brainstem volumes by 36%, of mesencephalon volumes up to 46%. Normalization with TICV or v-scale alone showed comparable results.

Brainstem volume normalization based on each of the three normalization models significantly reduced the inter-individual variability compared to the non-normalized volumes. Comparison between the three normalization models showed no significant differences in inter-individual variability of brainstem and brainstem substructure volumes, indicating an equal efficiency of normalization by these models.

TICV and v-scale are frequently applied normalization parameters for brain volumes because in general not affected by neurological/neurodegenerative diseases. By normalization with TICV, inter-individual variation of brain volumes was previously reduced about 4% (Whitwell et al., 2001). Using a similar methodological approach normalization with v-scale reduced variation in spinal cord volumetry by up to 10.24% (Papinutto et al., 2019).

By reducing measurement variability, we expect the proposed normalization methods to improve the sensitivity in detecting subtle brainstem volume differences between patients with diseases affecting the brainstem and/or its substructures and healthy controls or between patients' subgroups. Thus, previous studies showed improved detection of spinal cord volume differences between multiple sclerosis patients and controls after cervical volume normalization (Oh et al., 2014). Brainstem volume normalization, by reducing anatomical variability, might allow to reveal and strengthen clinical-radiological correlations in neurodegenerative diseases such as multiple sclerosis or Alzheimer's disease (Zhou et al., 2014).

The absence of brainstem volume reductions with increasing age observed in our cross-sectional study is in line with findings in other cross-sectional studies of Raz et al. (2001), Sullivan et al. (2004), and Walhovd et al. (2011). Walhovd et al. reported age-related volume differences in all examined brain structures except the brainstem based on FreeSurfer assessments in a large cohort of HS. To disentangle the exact mechanisms underlying the relative volume preservation of the brainstem with increasing age is beyond the scope of this descriptive study. As a phylogenetically relatively old structure the brainstem is crucial for survival. The reasons for its relative resilience to atrophy compared to other phylogenetically old structures like the hippocampus (Jack et al., 1998; Schröder and Pantel, 2016), amygdala (Kurth et al., 2019) and entorhinal cortex (Hasan et al., 2016) remain unknown. Potential limitations of this study include the underrepresentation of very advanced age and the cross-sectional design that does not allow intra-individual comparisons. Longitudinal studies covering a sufficiently long time-span are difficult to perform, but are certainly necessary to confirm our cross-sectional results in this regard.

The vital function of the brainstem, its clinical involvement in neurodegenerative and neuroinflammatory diseases, and the absence of volume reductions observed in HS aged from 18 to 72 years in this study render atrophy assessments of the brainstem and its substructures an interesting imaging surrogate candidate for the study of neurodegeneration as e.g., in progressive multiple sclerosis. This study analyzed different sources of both extrinsic and intrinsic variability of brainstem volumetry assessments and evaluated normalization models for variability reduction in healthy controls. The inter-individual anatomical variability of total brainstem volumes is relatively high but can be efficiently reduced by 36% using a normalization based on both TICV and age, and by about 34% based on TICV or v-scale alone.

This study's automated segmentation approach proved to be robust across different scanners, field strengths and imaging protocols and allows very fast, efficient, anatomically accurate, and reliable automated brainstem segmentation.

## Data Availability Statement

The data analyzed in this study is subject to the following licenses/restrictions: Upon reasonable request, we will render the detailed results derived from the reported analyses available. Requests to access these datasets should be directed to regina.schlaeger@usb.ch.

## Ethics Statement

The studies involving human participants were reviewed and approved by Ethikkommission Nordwest- und Zentralschweiz. The participants provided their written informed consent to participate in these studies.

## Author Contributions

LS: conceptualization, methodology, analysis, and writing. AH, SP, and SA: methodology and analysis. MA: data collection. TS: analysis. MW and EK: proof reading and analysis. ÖY: data collection and proof reading. LK and CG: proof reading and methodology. JW and PC: supervision, proof reading, and acquiring funding. RS: conceptualization, analysis, writing, supervision, and acquiring funding. All authors contributed to the article and approved the submitted version.

## Conflict of Interest

ÖY received grants from ECTRIMS/MAGNIMS, University of Basel, Pro Patient Stiftung, University Hospital Basel, Free Academy Basel, Swiss Multiple Sclerosis Society and advisory board fees from Sanofi Genzyme, Biogen, Almirall and Novartis. LK institution (University Hospital Basel) received in the last 3 years and used exclusively for research support at the Department: steering committee, advisory board, consultancy fees and support of educational activities from Actelion, Allergan, Almirall, Baxalta, Bayer, Biogen, Celgene/Receptos, CSL-Behring, Desitin, Excemed, Eisai, Genzyme, Japan Tobacco, Merck, Minoryx, Novartis, Pfizer, F. Hoffmann-La Roche Ltd, Sanofi Aventis, Santhera, and Teva, and license fees for Neurostatus-UHB products; the research of the MS center in Basel has been supported by grants from Bayer, Biogen, Novartis, the Swiss MS Society, the Swiss National Research Foundation, Inno-Suisse, the European Union, and Roche Research Foundations. CG the University Hospital Basel (USB), as the employer of CG has received the following fees which were used exclusively for research support: (i) advisory board and consultancy fees from Actelion, Novartis, Genzyme, and F. Hoffmann-La Roche; (ii) speaker fees from Biogen and Genzyme-Sanofi; (iii) research support by F. Hoffmann-La Roche Ltd. Before my employment at USB, I have also received speaker honoraria and travel funding by Novartis. JW was CEO of the MIAC AG Basel, Switzerland. He served on scientific advisory boards of Actelion, Biogen, Genzyme-Sanofi, Idorsia, Novartis, and Roche. He was supported by grants of the EU (Horizon2020), German Federal Ministries of Education and Research (BMBF) and of Economic Affairs and Energy (BMWI). RS was supported by the Swiss National Science Foundation (MHV program, PMPDP3 171391), the University of Basel, and the Swiss MS Society. The remaining authors declare that the research was conducted in the absence of any commercial or financial relationships that could be construed as a potential conflict of interest.
