# Supplementary material for: Improving Accuracy of Brainstem MRI Volumetry: Effects of Age and Sex, and Normalization Strategies
Source: Front Neurosci. 2020 Dec 23;14:609422. doi: 10.3389/fnins.2020.609422 (PMC7785816; doi:10.3389/fnins.2020.609422)
Supplement: Supplementary file 1 [file Data_Sheet_1.docx]

**APPENDIX**

**A**

|  | **Men**  **(unadjusted)** | **Women**  **(unadjusted)** | **Men**  **(adjusted for age and TICV)** | **Women**  **(adjusted for age and TICV)** |
| --- | --- | --- | --- | --- |
| **Brainstem**  (adj.) mean volume [mm^3^]  Standard error  95%CI  p (difference of the mean) | 28274.0 24826.2  385.6 358.5  27509.6- 24115.6-  29038.3 25536.9  **<0.0001** | | 26745.5 26147.7  336.1 307.2  26079.1- 25538.4-  27411.9 26756.5  0.2397 | |
| **Mesencephalon**  (adj.) mean volume [mm^3^]  Standard error  95%CI  p (difference of the mean) | 6863.5 6001.2  80.1 74.5  6704.8- 5853.6-  7022.3 6148.8  **<0.0001** | | 6517.7 6300.1  61.9 56.6  6394.9- 6188.0-  6640.4 6412.3  0.0215 | |
| **Pons**  (adj.) mean volume [mm^3^]  Standard error  95%CI  p (difference of the mean) | 16140.6 14334.4  265.9 247.2  15613.6- 13844.4-  16667.6 14824.3  **<0.0001** | | 15158.8 15183.0  243.0 222.1  14677.1- 14742.8-  15640.5 15623.3  0.9473 | |
| **Medulla oblongata**  (adj.) mean volume [mm^3^]  Standard error  95%CI  p (difference of the mean) | 5269.8 4490.7  64.3 59.8  5142.3- 4372.2-  5397.3 4609.2  **<0.0001** | | 5069.1 4664.2  63.9 58.4  4942.4- 4548.5-  5195.7 4780.0  **<0.0001** | |

**Table A1**: Mean volumes (with and without adjustment for TICV and age), standard error and 95%CI for total brainstem, mesencephalon, pons, and medulla oblongata for men and women (least square means). p-values surviving the Bonferroni correction <0.0125 are bolded.

**B** With adjustment for sex, there was no significant association between age and mesencephalon (p=0.3023), pons (p=0.1846), and medulla oblongata (p=0.4718) volumes, respectively. Alike, between older subjects (aged above the group mean of 35 years; n=44) and younger subjects (<35 years; n=66), brainstem substructure volumes did not differ significantly for mesencephalon (p=0.5336), pons (p=0.1662), and medulla oblongata (p=0.2331) (adjusted for sex).

**C** Adjusted for sex and field strength (1.5T vs. 3T), older subjects had no significant difference for mesencephalon (p=0.4898), pons (p=0.154), medulla oblongata (p=0.1353), and total brainstem (p=0.2779) volumes compared to younger subjects (<35 years).

Adjusted for sex and acquisition protocol, older subjects had no significant difference for mesencephalon (p=0.4258), pons (p=0.3014), medulla oblongata (p=0.2764), and total brainstem (p=0.4696) volumes compared to younger subjects (<35 years).

**D**

|  | **Mesencephalon** | | **Pons** | | **Medulla oblongata** | |
| --- | --- | --- | --- | --- | --- | --- |
| **Variable** | **P** | **Pearson**  **Corr.**  **Coeff.** | **P** | **Pearson**  **Corr.**  **Coeff.** | **P** | **Pearson**  **Corr.**  **Coeff.** |
| Nasion-opisthion | **<0.0001** | 0.538 | **<0.0001** | 0.369 | **<0.0001** | 0.565 |
| Dens length | **<0.0001** | 0.384 | **0.0003** | 0.342 | **<0.0001** | 0.444 |
| TICV | **<0.0001** | 0.839 | **<0.0001** | 0.673 | **<0.0001** | 0.691 |
| v-scale | **<0.0001** | -0.796 | **<0.0001** | -0.676 | **<0.0001** | -0.722 |
| Age | 0.844 | -0.019 | 0.100 | 0.158 | 0.222 | 0.117 |
| WM volume | **<0.0001** | 0.835 | **<0.0001** | 0.717 | **<0.0001** | 0.754 |
| GM volume | **<0.0001** | 0.693 | **<0.0001** | 0.472 | **<0.0001** | 0.542 |
| BV | **<0.0001** | 0.819 | **<0.0001** | 0.638 | **<0.0001** | 0.695 |
| Basion-opisthion | **0.0015** | 0.300 | 0.0931 | 0.161 | **0.0002** | 0.343 |
| Foramen magnum diameter | 0.1779 | 0.129 | 0.695 | -0.038 | 0.008 | 0.252 |
| Dens-  opisthion | **0.0004** | 0.334 | 0.0069 | 0.256 | **<0.0001** | 0.455 |
| Brainstem  angle | 0.0312 | 0.206 | 0.0128 | 0.237 | 0.0157 | 0.230 |

**Table D.1**: Pearson correlation coefficients of all metrics and brainstem substructure volumes. p-values surviving the Bonferroni correction <0.0042 are bolded.

**E**

|  | **Mesencephalon** | | | **Pons** | | | **Medulla oblongata** | | |
| --- | --- | --- | --- | --- | --- | --- | --- | --- | --- |
|  | **p** | **Adj. r^2^** | **estimate** | **p** | **Adj. r^2^** | **estimate** | **p** | **Adj. r^2^** | **estimate** |
| **Model 1a:**  TICV  Age | <0.0001  <0.0001  0.3222 | 0.700 | 0.0036  2.9307 | <0.0001  <0.0001  0.0021 | 0.490 | 0.0087  35.5148 | <0.0001  <0.0001  0.0106 | 0.500 | 0.0026  8.3367 |
| **Model 1b:**  TICV | <0.0001  <0.0001 | 0.701 | 0.0036 | <0.0001  <0.0001 | 0.447 | 0.0085 | <0.0001  <0.0001 | 0.473 | 0.0025 |
| **Model 2:**  v-scale | <0.0001  <0.0001 | 0.630 | -4149.025 | <0.0001  <0.0001 | 0.452 | -10333.96 | <0.0001  <0.0001 | 0.517 | -3167.896 |

**Table E.1:** Linear regression analysis with mesencephalon, pons, and medulla oblongata volumes as outcomes, respectively.

**F**

|  | **Non-normalized** | **Model 1a** | **Model 1b** | **Model 2** |
| --- | --- | --- | --- | --- |
| **Mesencephalon**  %RSD  Relative %RSD reduction [%]  Mean % inter-individual variability  SD | 11.17  9.11  6.40 | 6.05  45.84  4.92  3.49 | 6.08  45.57  4.90  3.57 | 6.76  39.48  5.40  4.04 |
| **Pons**  %RSD  Relative %RSD reduction [%]  Mean % inter-individual variability  SD | 13.81  11.20  8.01 | 9.78  29.18  7.91  5.70 | 10.22  26.00  8.23  6.00 | 10.18  26.29  8.25  5.91 |
| **Medulla oblongata**  %RSD  Relative %RSD reduction [%]  Mean % inter-individual variability  SD | 12.39  10.01  7.23 | 8.68  29.94  7.03  5.05 | 8.95  27.76  7.26  5.20 | 8.57  30.83  6.83  5.13 |

**Table F.1:** % relative standard deviation (RSD, standard deviation divided by the mean volume), relative %RSD reduction, mean % inter-individual variability ((measured volume – mean volume) / mean volume), and SD of the % inter-individual variability with respect to the measured mesencephalon, pons, and medulla oblongata volumes for normalizations based on models 1a, 1b, 2.
